# PRMT6 physically associates with nuclear factor Y to regulate photoperiodic flowering in *Arabidopsis*

**DOI:** 10.1007/s42994-021-00065-y

**Published:** 2021-12-02

**Authors:** Pingxian Zhang, Xiulan Li, Yifan Wang, Weijun Guo, Sadaruddin Chachar, Adeel Riaz, Yuke Geng, Xiaofeng Gu, Liwen Yang

**Affiliations:** 1 Biotechnology Research Institute, Chinese Academy of Agricultural Science, Beijing, 100081 China; 2 College of Life Science and Technology, Huazhong Agricultural University, Wuhan, 430070 Hubei China; 3 College of Life and Environmental Sciences, Minzu University of China, Beijing, 100081 China

**Keywords:** Arginine methylation, Protein arginine methyltransferases 6, Nuclear factors Y, Flowering time

## Abstract

The timing of floral transition is critical for reproductive success in flowering plants. In long-day (LD) plant *Arabidopsis*, the floral regulator gene *FLOWERING LOCUS T* (*FT*) is a major component of the mobile florigen. *FT* expression is rhythmically activated by CONSTANS (CO), and specifically accumulated at dusk of LDs. However, the underlying mechanism of adequate regulation of *FT* transcription in response to day-length cues to warrant flowering time still remains to be investigated. Here, we identify a homolog of human protein arginine methyltransferases 6 (HsPRMT6) in *Arabidopsis*, and confirm AtPRMT6 physically interacts with three positive regulators of flowering Nuclear Factors YC3 (NF-YC3), NF-YC9, and NF-YB3. Further investigations find that *AtPRMT6* and its encoding protein accumulate at dusk of LDs. PRMT6-mediated H3R2me2a modification enhances the promotion of NF-YCs on *FT* transcription in response to inductive LD signals. Moreover, AtPRMT6 and its homologues proteins AtPRMT4a and AtPRMT4b coordinately inhibit the expression of *FLOWERING LOCUS C*, a suppressor of *FT*. Taken together, our study reveals the role of arginine methylation in photoperiodic pathway and how the PRMT6-mediating H3R2me2a system interacts with NF-CO module to dynamically control *FT* expression and facilitate flowering time.

**Supplementary Information:**

The online version contains supplementary material available at 10.1007/s42994-021-00065-y.

## Introduction

Floral transition is controlled by several exogenous and endogenous developmental signals to ensure appropriate flowering time. To date, this transition is regulated by the photoperiodic, vernalization, autonomous, gibberellin, and the miR156-SPL module-mediated aging pathways (Wang et al. 2009; Andrés and Coupland 2012; He 2012; Bouché et al. 2017), in which the day length (photoperiod) is a stable seasonal signal to guarantee optimal flowering time in plants (Andrés and Coupland 2012; Romera-Branchat et al. 2014). *Arabidopsis thaliana*, a facultative long-day (LD) plant, can respond to photoperiodic signals to control floral transition by modulating the transcription of *FLOWERING LOCUS T* (*FT*) specifically in leaf vasculature via CONSTANS (CO)-dependent pathway (Andrés and Coupland 2012; Song et al. 2015). The B and C subunits of Nuclear Factor Y (NF-YB and NF-YC), as histone fold domain (HFD) NF-YB/NF-YC dimer, directly associate with CO thus compose of a trimeric NF-CO complex to regulate the *FT* expression (Wenkel et al. 2006; Gnesutta et al. 2018). Two NF-YB subunits (NF-YB2 and NF-YB3), and three NF-YC subunits (NF-YC3, NF-YC4, and NF-YC9) have been shown to interact with CO to activate *FT* expression (Kumimoto et al. 2010; Cao et al. 2014). Subsequently, FT protein, a major component of mobile florigen, transports from leaf veins to the shoot apex and leads to the floral transition (Andrés and Coupland 2012; Liu et al. 2013; Putterill and Varkonyi-Gasic 2016).

Except for the NF-CO complex, another regulator of *FT* expression is Polycomb group (PcG) which function to silence the transcription of *FT* (Wang et al. 2014; Müller-Xing et al. 2014; Luo et al. 2018). PcG proteins contain Polycomb repressive complex 1 (PRC1) and PRC2. PRC2 acts as a methyltransferase complex to catalyze histone 3 lysine-27 trimethylation (H3K27me3) on target chromatin, while PRC1 has been shown to maintain the H3K27me3 mark, leading to additional transcription repression by repressive chromatin modifications (Mozgova and Hennig 2015; Förderer et al. 2016). In *Arabidopsis*, two plant-unique BAH (Bromo adjacent homology) proteins SHORT LIFE (SHL) and EARLY BOLTING IN SHORT DAYS (EBS) form with a complex with EMBRYONIC FLOWER 1 (EMF1) to inhibit *FT* expression by reading the repressive H3K27me3 marks on *FT* locus (Li et al. 2018; Yang et al. 2018; Jing et al. 2019a). As another reader of H3K27me3, the LIKE HETEROCHROMATIN PROTEIN 1 (LHP1) has been shown to maintain this mark on *FT* locus (Turck et al. 2007; Zhang et al. 2007), and LHP1 can also directly interact with EMF1 to silence *FT* expression and thus to repress photoperiodic flowering (Wang et al. 2014). Remarkably, the NF-CO module is partly co-activated to relieve Polycomb repression on the transcription of *FT*, resulting in the de-repression of *FT* that confer the LD induction of floral transition in *Arabidopsis* (Luo et al. 2018). In addition, other chromatin modifications such as histone deacetylation SIN3 LIKEs (SNLs) and chromatin remodeler PICKLE (PKL) are involved in the regulation of flowering by facilitating *FT* expression at dusk (Huang et al. 2019; Jing et al. 2019b). Although these studies have emphasized the significance of epigenetic regulation in photoperiodic flowering, it is still to be established whether and how other chromatin modifiers ‘communicate’ with NF-CO and NF-Y in response to inductive LDs to modulate *FT* expression.

Protein arginine methyltransferases (PRMTs) are responsible for catalyzing methylation at arginine (R) residues on histones that utilize S-adenosyl-L-methionine as a methyl donor. Besides, PRMTs-catalyzed non-histone substrates are involved in various biological processes including gene transcription, RNA processing and transport, cell signaling, DNA repair, and cell differentiation in mammals (Blanc and Richard 2017; Neault et al. 2012; Stein et al. 2012; Damez-Werno et al. 2016). In *Arabidopsis*, the posttranslational modifications of R mainly occur on histone 3 (H3) at R2, R8, R17, R26, and on H4 at only R3, and these modifications are catalyzed by different PRMTs (Ahmad and Cao 2012). PRMTs are divided into type I, comprising AtPRMT1a, AtPRMT1b, AtPRMT3, AtPRMT4a, AtPRMT4b, and AtPRMT6; type II, comprising AtPRMT5; type III, comprising only AtPRMT7; and a plant-specific AtPRMT10 (Ahmad and Cao 2012). These different types of PRMTs are responsible for producing symmetric *ω*-*N*^*G*^-monomethyl arginine (MMA), asymmetric *ω*-*N*^*G*^,*N*^*G*^-dimethylarginine (aDMA), and symmetric ω-*N*^*G*^,*N*^*G*^-dimethylarginine (sDMA) (Zurita-Lopez et al. 2012). Several studies indicate that PRMT4a/4b, PRMT5, and PRMT10 mediate histone arginine methylation to regulate flowering time by repressing the *FLOWERING LOCUS C (FLC)* expression (Niu et al. 2007, 2008; Pei et al. 2007; Schmitz et al. 2008). However, whether PRMTs involve in the regulation of photoperiodic *FT* expression remains unknown.

In this study, we identify a positive regulator of flowering PRMT6, which has been shown to catalyze the asymmetric dimethylation of R2 on H3 (H3R2me2a) (Guccione et al. 2007; Hyllus et al. 2007; Iberg et al. 2008). Further investigation finds that *PRMT6* gene and its encoding protein accumulate in leaf veins at dusk, suggesting that PRMT6 may be associated with *FT* gene. PRMT6 interacts with NF-YCs and enhances their promotion on *FT* transcription. Moreover, AtPRMT6 and its homologues proteins AtPRMT4a and AtPRMT4b coordinately inhibit the expression of *FLC*, a suppressor of *FT*. Our results uncover the function of PRMT6 in plants, and provide insight into the role of arginine methylation in regulating photoperiodic flowering by ‘communicating’ with transcription factors.

## Results

### PRMT6 interacts with NF-Y subunits

**Fig. 1:**
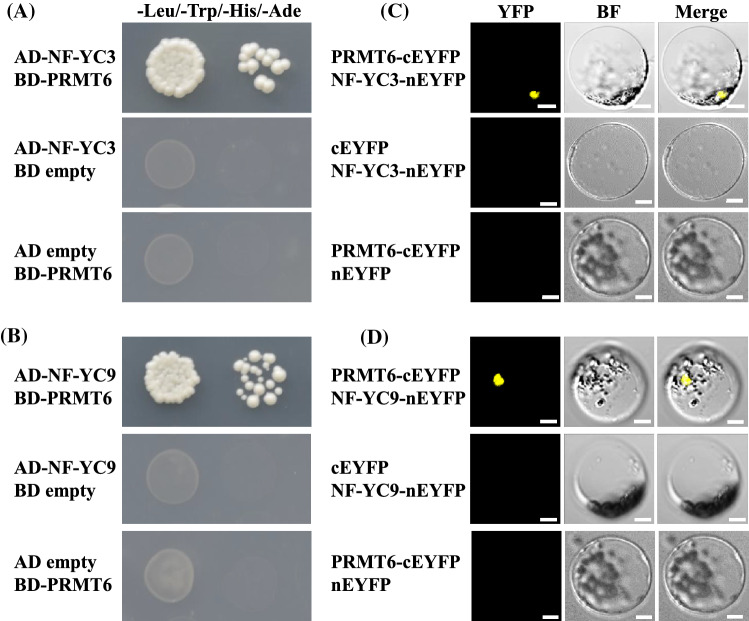
Direct interactions of AT3G20020 (PRMT6) with NF-YC3 and NF-YC9 proteins. **A**, **B** Interactions of PRMT6 with NF-YC3 (**A**) and NF-YC9 (**B**) in yeast. Yeast cells harboring protein fusions with the DNA-binding domain (BD) and/or activation domain (AD) as indicated were grown on selective synthetic defined media lacking Trp, Leu, and His. **C**, **D** BiFC analysis of the interactions of PRMT6 with NF-YC3 (**C**) and NF-YC9 (**D**) in *Arabidopsis* protoplasts. *Arabidopsis* protoplasts were co-transformed transiently by a pair of plasmids. Yellowish-green signals indicate physical associations of paired proteins in the nuclei. Bar = 10 µm

Two subunits of NF-Y proteins (NF-YBs and NF-YCs) have been reported to interact with the CO and form NF-CO complex to regulate photoperiodic flowering (Hou et al. 2014; Gnesutta et al. 2018). We observed that two NF-YC members, NF-YC3 and its homolog NF-YC9, could interact with PROTEIN ARGININE METHYLTRANSFERASE 6 (AT3G20020; PRMT6) in yeast cells (Fig. 1A, B). Additionally, PRMT6 also strongly interacted with NF-YB3 in yeast cells, but did not interact with NF-YB2 and NF-YC4 (Fig. S1a). Next, we performed in vivo bimolecular fluorescence complementation (BiFC) assay to verify these interactions. The enhanced yellow fluorescent protein (EYFP) with non-fluorescent N-terminal was fused to the full-length CDS of NF-YC3, NF-YC9, and NF-YB3, and the C-terminal fragments were fused to PRMT6. When PRMT6-cEYFP and NF-YC3-nEYFP were co-expressed in *Arabidopsis* mesophyll protoplasts, the fluorescence was observed in nucleus, but not from protoplasts co-expressing NF-YC3-nEYFP and cEYFP alone, or PRMT6-cEYFP and nEYFP alone (Fig. 1C). Similarly, we also confirmed the interactions of PRMT6 with NF-YC9 (Fig. 1D), and PRMT6 with NF-YB3 (Fig. S1a, b). Concomitantly, a transient coimmunoprecipitation (Co-IP) assay was performed to testify the direct interaction of PRMT6 and NF-YC3 (Fig. S1c). We subsequently explored whether PRMT6 could physically associate with CO or FT protein, and found that PRMT6 did not interact with CO or FT in yeast cells (Fig. S1a).

### *PRMT6 *enhances the promotion of *NF-YC3;4;9* on flowering by facilitating *FT* expression

**Fig. 2:**
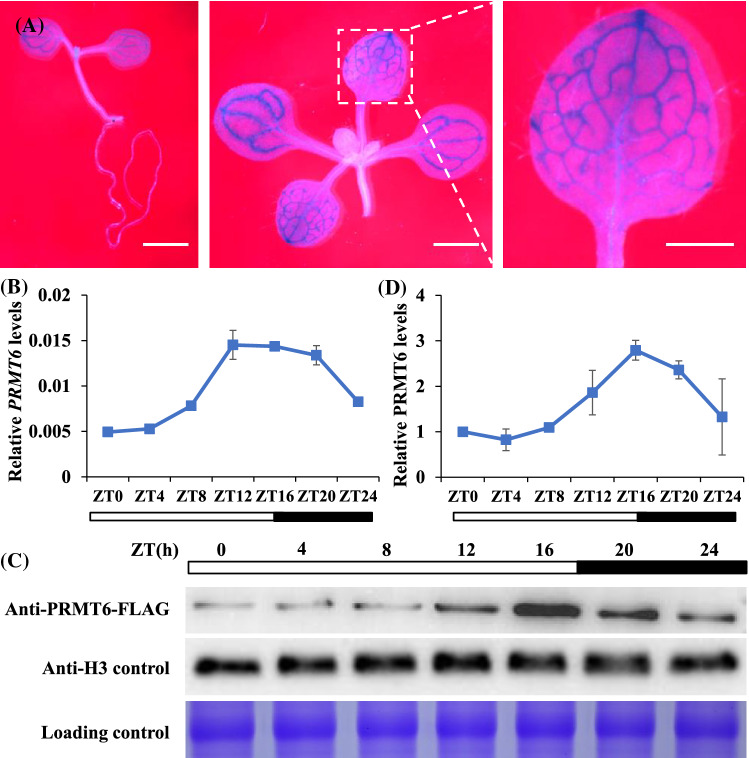
*PRMT6* diurnally expressed at dusk in the vascular bundle cells under LDs. **A** Spatial expression patterns of *PRMT6-GUS* in 5-day-old seedlings, and 10-day-old seedlings of aerial part and cotyledon. Plants were stained for 6 h. Scale bars = 1 mm. **B** The relative transcription level of *PRMT6* in 10-day-old Col seedlings under LDs. The transcription levels were normalized to *UBQ10*, and relative fold changes to Zeitgeber time 0 (ZT0) are presented. Bars indicate s.d. of triplicate measurements. White and dark bars below the *x*-axis indicate light and dark periods, respectively. **C**, **D** The expression levels of PRMT6-FLAG protein over a 24-h LD cycle examined by western blotting. Total proteins loaded in SDS-PAGE gels were stained with Coomassie Blue, antibody or the relative PRMT6-FLAG protein levels were normalized to H3 by the ImageJ program (**D**). The error bars indicate the s.d. measurements

Analyses of the *GUS* reporter expression in transgenic plants expressing GUS under control of the *PRMT6* promoter indicated that *PRMT6* promoter was active in the vascular bundle cells (Fig. 2A). Subsequently, we measured the *PRMT6* expression pattern every 4 h over a 24 h LD cycle in Col. *PRMT6* mRNA abundance increased during daylight, peaks at ZT16 and then decreased (Fig. 2B). Additionally, we constructed a *PRMT6-FLAG* expression line driven by the native promoter region of *PRMT6* and measured the protein abundance in the *PRMT6-FLAG* lines. PRMT6 protein varied diurnally and also peaked at ZT16 under LD conditions (Fig. 2C, D). Subcellular localization analysis showed that PRMT6 is a nuclear protein (Fig. S2). Collectively, nucleic protein PRMT6 displayed diurnal expression pattern and accumulated at dusk under LDs, which was consistent with the previously reported expression pattern of *FT-GUS* (Gu et al. 2013) and indicated that PRMT6 might modulate flowering by regulating *FT* transcription.

**Fig. 3:**
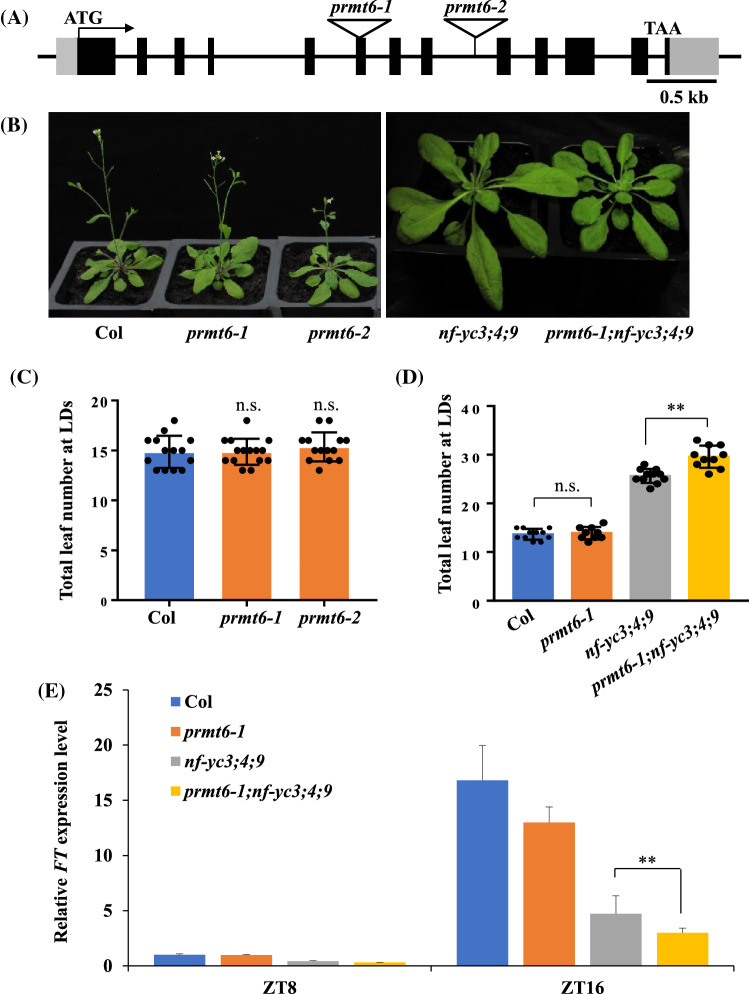
Loss of *PRMT6* function delays the floral transition of *nf-yc3;4;9* by decreasing the *FT* expression under LDs. **A** Gene structure of *PRMT6*. Exons and 5’ untranslated region (UTR) or 3’ UTR are represented by black boxes and gray boxes, and arrows indicate transcription start sites (TSS); the T-DNA insertion sites of two lines are indicated with triangles. **B** Phenotype of Col, *prmt6-1*, *prmt6-2*, *nf-yc3;4;9*, and *prmt6-1;nf-yc3;4;9* mutants grown in LDs. **C** Flowering times of the indicated genotypes grown in LDs. More than ten plants for each line were scored; bars indicated for standard deviation (s.d.); n.s. indicated non-significant difference. **D** Flowering times of the indicated lines grown in LDs. More than ten plants for each line were scored; bars indicated for s.d.; n.s. indicated non-significant difference; Double asterisks indicated statistically significant differences in the means between the indicated genotypes, as revealed by two-tailed Student’s *t* test (**p < 0.01). **E** Relative *FT* transcript levels in the seedlings of the indicated genotypes grown in LDs at ZT8 and ZT16. The transcript levels were first normalized to that of *UBQ10*. Bars indicate the s.d. of triplicate measurements

Next, two transfer DNA (T-DNA) insertion single-mutant *prmt6-1* (Sail_385_A06) and *prmt6-2* (Salk_151679C) (Figs. 3A; S3a) were used to explore its biological function in flowering. The total number of leaves of *prmt6-1* and *prmt6-2* mutant are not significantly different with Col under LD and SD conditions (Figs. 3B, C, S3b). To further confirm whether *PRMT6* overexpression contributed to flowering time, we constructed overexpression lines and also found no significant differences between the Col and *PRMT6* overexpression lines under LD conditions (Fig. S3c). We further explore whether *PRMT6* mutation could affect the flowering phenotype of *nf-yc3;4;9* mutants, and crossed *prmt6-1* mutant with the *nf-yc3;4;9* triple mutant to produce the quadruple mutant *prmt6-1;nf-yc3;4;9*. The total number of leaves in the *prmt6-1;nf-yc3;4;9* was significantly more than that of *nf-yc3;4;9* (Fig. 3B, D), suggesting that *PRMT6* mutation could delay the flowering time of *nf-yc3;4;9* mutant. Moreover, the expression of *FT* in *prmt6-1;nf-yc3;4;9* mutant was lower than that of the *nf-yc3;4;9* triple mutant line at ZT16 under LDs (Fig. 3E), in accordance with the additive action of *prmt6-1* to delay flowering of *nf-yc3;4;9*.

### PRMT6 directly binds on the *FT* locus and affects its H3R2me2a levels

Because PRMT6 homologs have been identified to catalyze H3R2me2a in mammals (Guccione et al. 2007; Hyllus et al. 2007; Iberg et al. 2008), we performed sequence alignment of PRMT6 homologs in human, mouse, zebrafish, and *Arabidopsis* to test whether *Arabidopsis* PRMT6 have sequence similarity with PRMT6 homologs in animals. AtPRMT6 protein contained conserved domains including AdoMet methyltransferase (MTase) I, post-I, II, and III as well as the THW loop, which were involved in R methyltransferase activity (Fig. S4a, b).

**Fig. 4:**
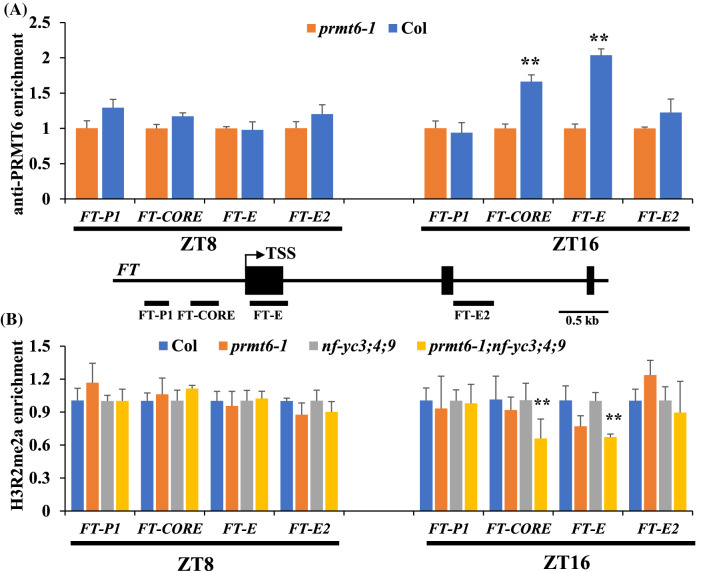
PRMT6 modulates H3R2me2a methylation level and its binding on *FT* chromatin at dusk under LDs. **A** PRMT6 enrichment at the *FT* locus at midday (ZT8) and dusk (ZT16) under LDs. PRMT6 antibody was used to immunoprecipitate target chromatin extracted from Col or *prmt6-1* (served as control). The amounts of immunoprecipitated genomic fragments were measured by RT-qPCR, and subsequently normalized to *TUBLIN8* (*TUB8*). Error bars indicate s. d. from three technical replications. The regions examined in ChIP experiments are indicated with solid lines under FT structure. **B** H3R2me2a levels at the *FT* locus at dusk (ZT16) under LDs. The amounts of immunoprecipitated genomic fragments were quantified, and subsequently normalized to the input DNA. Error bars indicate s.d.

We then examined whether the *PRMT6* mutation could affect the global H3R2me2a levels. Histones were extracted from 10-day-old seedlings and probed with antibodies against H3R2me2a and H3. The global levels of H3R2me2a in *prmt6-1* were similar to these in Col (Fig. S5a). Next, we performed chromatin immunoprecipitation (ChIP) assays to explore whether PRMT6 could bind on *FT* locus and affect its H3R2me2a methylation level. First, we constructed a PRMT6 antibody and confirmed its specificity (Fig. S5b). ChIP assays were conducted using 10-day-old *prmt6-1* and Col seedlings grown under LDs. PRMT6 was enriched at the *FT* promoter region at ZT16 but not at ZT8 in Col compared to *prmt6-1* mutant (Fig. 4A). Considering the *NF-YCs’* transcript exhibits photoperiodic expression pattern under LD conditions (Fig. S6), we further performed ChIP experiments in Col, *prmt6-1*, *nf-yc3;4;9*, and *prmt6-1;nf-yc3;4;9* lines at ZT8 and ZT16 of LDs, to verify whether PRMT6 could affect the H3R2me2a level to *FT* chromatin in the *nf-yc3;4;9* mutant background. The H3R2me2a methylation level at *FT* locus of the *prmt6-1; nf-yc3;4;9* quadruple mutant was significantly reduced at ZT16 but not at ZT8 (Fig. 4B). Collectively, these results demonstrated that PRMT6 mediated H3R2me2a modification to modulate *FT* transcription at the end of daylight.

### PRMT6 functions redundantly with PRMT4a and PRMT4b in the *Arabidopsis* genome

**Fig. 5:**
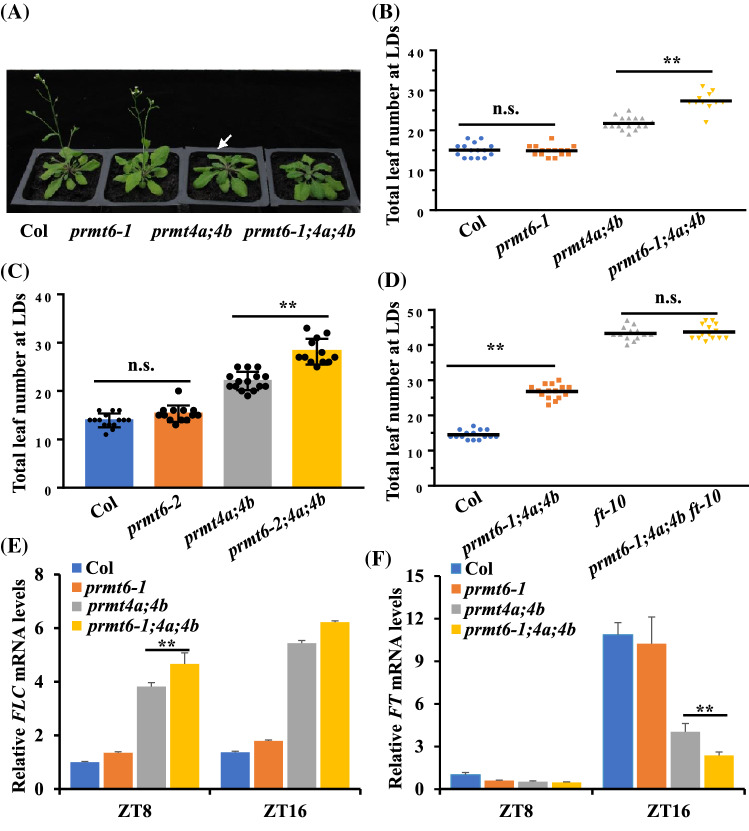
*PRMT6* mutation enhances the late flowering phenotype of *prmt4a;4b* double mutant. **A** Phenotypes of Col, *prmt6-1*, *prmt4a;4b,* and *prmt6-1;4a;4b* mutants. Plants were grown under LD conditions. The white arrow indicates that the *prmt4a;4b* double mutants were bolting. **B** Flowering time of Col, *prmt6-1*, *prmt4a;4b*, and *prmt6-1;4a;4b* under LD conditions. More than 15 plants for each line were scored; bars indicate the s.d.; significant differences between the means of the indicated genotypes were revealed by a two-tailed Student’s *t* test (n.s., no significant difference; *p < 0.05; **p < 0.01). **C** Flowering time of Col, *prmt6-2*, *prmt4a;4b*, and *prmt6-2;4a;4b* grown under LD conditions. **D** Flowering time of Col, *prmt6-1;4a;4b*, *ft-10*, and *prmt6-1;4a;4b ft-10* grown under LD conditions. **E**, **F** the relative transcription levels of *FLC* (**E**) and *FT* (**F**) in seedlings of the indicated genotypes as quantified by RT-qPCR. The transcription levels were normalized to *UBQ10*, and the fold changes relative to Col are presented

Previously, AtPRMT4a/4b (type I PRMT proteins) were shown to play important roles in regulating flowering time by influencing the transcription of *FLC*, which involved in vernalization and autonomous pathways (Niu et al. 2008). To clarify the genetic interaction between PRMT6 and PRMT4a/4b in regulating floral transition, we then generated *prmt6-1;4a;4b* and *prmt6-2;4a;4b* triple mutants by crossing. And the homozygous *prmt6-1;4a;4b* were used for subsequent analysis. The total leaf number of the *prmt6-1;4a;4b* and *prmt6-2;4a;4b* was more than *prmt4a;4b* double mutant, indicating that *prmt6;4a;4b* triple mutant exhibited the delayed flowering phenotype compared to *prmt4a;4b* double mutant under LD conditions (Fig. 5A–C). Next, we generated *prmt6-1;4a;4b;ft-10* quadruple mutant lines to determine whether the *ft-10* mutation could rescue the *prmt6-1;4a;4b* triple mutant phenotype. As expected, we found that the quadruple mutant line displayed a similar number of total leaves to the *ft-10* single mutant (Fig. 5D), suggesting that the genetic mechanism regulating the *prmt6-1;4a;4b* phenotype could be completely rescued by the *ft-10* mutation and that *FT* could thus be considered their downstream gene. Further investigation showed that the expression level of *FLC* in *prmt6;4a;4b* triple mutant was significantly higher than that in *prmt4a;4b* double mutant at ZT8 and ZT16 under LD conditions (Fig. 5E). *PRMT6* mutation only inhibited the *FT* mRNA abundance in *prmt6;4a;4b* triple mutant at ZT16 under LD conditions (Fig. 5F). Together, these findings revealed that PRMT6 may be not only involved in regulation of photoperiodic *FT* expression through NF-CO module, but also exhibits redundancy with PRMT4a/PRMT4b to regulate *FLC* expression, thus to promote floral transition in *Arabidopsis*.

## Discussion

### PRMT6 serves as co-factor of NF-YCs and PRMT4s during flowering

In animal systems, PRMT6 has been reported to play an important role in the regulation of disease and developmental processes (Boulanger et al. 2005; Yang and Bedford 2013), and acts as writers to catalyze H3R2me2a (Guccione et al. 2007; Hyllus et al. 2007; Iberg et al. 2008). However, the function of PRMT6 of regulating plant development remains unclear. In this study, we have first illustrated the involvement of AtPRMT6, an *Arabidopsis* homolog of human HsPRMT6, in the flowering transition. In *Arabidopsis prmt6* mutants exhibited a similar phenotype to wild type, whereas PRMT6 mutation further delays the flowering time of *prmt4a;4b* (Fig. 5; Niu et al. 2008), suggesting PRMT6 could function redundantly with PRMT4a/PRMT4b in controlling floral transition. In addition, our results indicated that the *PRMT6* mutation also strengthened the late flowering phenotype of *nf-yc3;4;9* (Fig. 3). These results demonstrated PRMT6, which might serve as a co-factor of NF-YCs and PRMT4s, synergistically modulated floral transition.

### PRMT6 control floral transition via NF-Y-CO module or *FLC*-dependent pathway

In *Arabidopsis*, movement of the FT protein (also known as florigen) contributes to inducing the flowering transition through long-distance signaling from the leaf vascular tissue (phloem) to the SAM (Corbesier et al. 2007), and *FT-GUS* was mainly expressed in the leaf vascular tissues (Gu et al. 2013). Our study found that *PRMT6* exhibited a similar expression pattern to that of *FT* in the leaf phloem (Fig. 2A, B). Moreover, the expression patterns of Flag-PRMT6 also showed rhythmic oscillations under LD conditions and accumulated at dusk under LDs (Fig. 2C), coinciding with the peak in CO protein level and increasing *FT* expression. These results demonstrated that PRMT6 could regulate CO-*FT* module. The nuclear factor NF-Y could bind to target DNA sequences accumulated on silent chromatin regions and act as a ‘pioneer’ to open up the chromatin structure to activate gene expression (Fleming et al. 2013; Oldfield et al. 2014). In addition, its subunits have been reported to interact with the CO protein (forming NF-CO complexes) to regulate flowering time (Gnesutta et al. 2018). In our study, PRMT6 was associated with NF-YC proteins, but did not interact with CO in yeast cells (Fig. S1). Considering the interaction between NF-YC proteins with CO testified by the previous studies (Hou et al. 2014; Gnesutta et al. 2018), we suppose that PRMT6 could interplay with CO by NF-YC proteins. Moreover, we confirmed the occupancy of PRMT6 at *FT* loci, as well as the reduced H3R2me2a modification in *prmt6-1;nf-yc3;4;9* compared with *nf-yc3;4;9* (Fig. 4). These data demonstrated that NF-YC proteins could recruit PRMT6 to *FT* promoter, consequently catalyzing H3R2me2a modification and finally accelerate floral transition. On the other hand, we observed PRMT6 and PRMT4 proteins synergistically inhibited the expression of *FLC* (Fig. 5). Taken together, PRMT6, NF-YCs, and PRMT4s synergistically modulated floral transition by CO-*FT* module or FLC-related pathway.

**Fig. 6:**
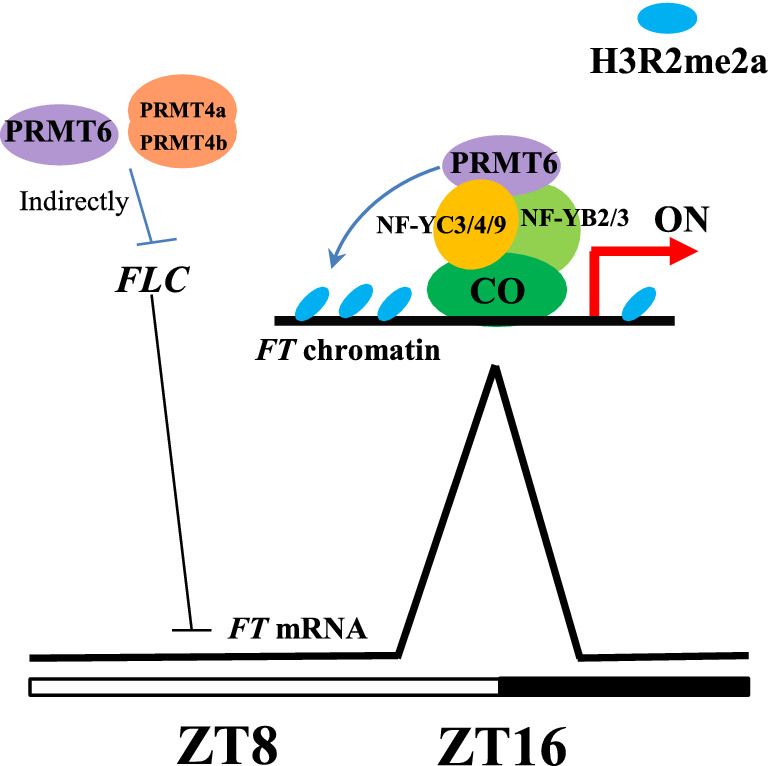
A working model for PRMT6 promoting flowering time in *Arabidopsis*. AtPRMT6 physically associates with three NF-Y subunits to bind to the *FT* locus around dusk (ZT16) of LDs, consequently changing the methylation abundance on *FT* locus to promote its expression at ZT16 of LDs. In addition, PRMT6 play a redundant role with PRMT4a/PRMT4b via inhibiting *FLC* expression during floral transition

In conclusion, our study revealed that AtPRMT6, a PRMT6 homolog in *Arabidopsis*, acts as a positive regulator of floral transition. AtPRMT6 physically associates with three NF-Y subunits to bind to the *FT* locus around dusk (ZT16) of LDs, consequently changing the methylation abundance on *FT* locus to promote its expression at ZT16 of LDs. In addition, PRMT6 play a redundant role with PRMT4a/PRMT4b via inhibiting *FLC* expression during floral transition (Fig. 6). Our study reveals the role of arginine methylation in photoperiodic pathway and how the PRMT6-mediating H3R2me2a system interacts with NF-CO module to dynamically control *FT* expression and facilitate flowering time.

## Materials and methods

### Plant materials, growth conditions, and flowering time

The *ft-10* (Kardailsky et al. 1999), *prmt4a;4b* (Niu et al. 2008), and *nf-yc3;4;9* (Hou et al. 2014) mutants were described previously. Two *prmt6* lines, *prmt6-1* (Sail_385_A06) and *prmt6-2* (Salk_151679C), were obtained from the ABRC. Plants were grown in LD conditions (16-h light/8-h dark) or SD conditions (8-h light/16-h dark) under cool white fluorescent light at 22 °C. Total leaf numbers (rosette and cauline leaves) were calculated as a measurement of flowering time. Approximately 15 plants were analyzed for each line.

### RNA extraction and RT-qPCR

Total RNA was extracted from the aerial parts of 10-day-old seedlings grown under LD conditions using the Qiagen RNeasy Plus Mini Kit and then reverse-transcribed into cDNA with M-MLV reverse transcriptase and oligo (dT) primers (Promega). Real-time quantitative PCR (RT-qPCR) was performed using the Roche Light Cycler 480 II System using SYBR Green PCR master mix, as described previously (Gu et al. 2013). Each sample was quantified at least three times and normalized to *UBQ10*. The relative transcription levels was calculated as 2^−△Ct^ (Gu et al. 2013). The primer pairs for *FT*, *NF-YC3*, *NF-YC9*, *TUB2*, and *UBQ10* amplification were described previously (Gu et al. 2013; Hou et al. 2014).

### Plasmid construction and plant transformation

To generate the *pPRMT6-PRMT6:FLAG* plasmid, the full-length *PRMT6* CDS without the stop codon was first fused with a *3* × *FLAG* tag and cloned into *pHGW* vector via Gateway technology. The sequences of the primers used for plasmid construction are specified in Table S1. *Agrobacterium tumefaciens* GV3101 was used to generate stable transgenic lines.

### Histochemical *β*-glucuronidase staining

To construct *AtPRMT6-GUS*, 4.5 kb genomic fragments including the 2.4 kb promoter and 2.1 kb genomic coding sequence of *PRMT6* (including exons and introns) were inserted into the *pMDC162* vector via Gateway technology (Invitrogen). Histochemical *β*-glucuronidase (GUS) staining was performed as described previously (Gu et al. 2013). Briefly, GUS staining was carried out with 5- and 10-day-old seedlings after hygromycin selection by immersing them in X-Gluc (5-bromo-4-chloro-3-indoyl β-d-glucuronide) (0.5 mg/ml) (Gold Biotechnology; USA), followed by vacuum briefly to ensure uniform staining. The tissues were then incubated in X-Gluc at 37 °C for various times followed by incubation in 70% ethanol at 37 °C overnight to remove the chlorophyll from the green tissues.

### Subcellular localization

For *PRMT6–GFP* fusion protein construction, the 1.3 kb CDS (coding sequence) region containing all the exons of *PRMT6* was inserted between the 35S promoter and *GFP* in the *pMDC85-GFP* vector via Gateway technology (Invitrogen). The subcellular localization of PRMT6 was conducted as described previously (Gu et al. 2013). GFP fluorescence signals were observed and recorded using a Zeiss LSM 700 confocal laser scanning microscope.

### Preparation of polyclonal antibody

The synthetic PRMT antibody was performed as described previously (Geng et al. 2020; Zhang et al. 2021). Briefly, a 15-amino acid fragment of PRMT6 (residues 105–119; TYREAIMQHQSLIEG) was synthesized by the solid-phase peptide synthesis (SPPS) method and confirmed by mass spectrometry (MS) and high-performance liquid chromatography (HPLC). The polypeptide was coupled through the Sulfo-SMCC agent to react with keyhole limpet hemocyanin (KLH) for preparation of the immune complex. The prepared immune complex was immunized to two rabbits to produce the antibody and then purified by a specific affinity chromatography column. The synthesized antibody was verified by western blot analysis using total proteins extracted from wild-type and *prmt6-1* plants.

### Histone extraction and immunoblotting

Histone protein extraction and western analysis were performed as described previously (Zhang et al. 2021). Briefly, total histones were extracted from 10-day-old seedlings grown in LD conditions, separated on an SDS-PAGE gel, and subsequently transferred to a 0.2-μm nitrocellulose membrane (Bio-Rad). The protein blots were probed with anti-H3 (Abcam, Cat#: ab1791) and H3R2me2a (Abcam, Cat#: ab175007). Immunoblotting was visualized by chemiluminescence. Blotting signals were captured using ImageJ software, and the relative protein level of H3R2me2a was normalized to that of H3. Experiments were repeated at least two biological times.

### Yeast two-hybrid (Y2H) assay

Yeast two-hybrid assays were conducted using the Matchmaker GAL4 Two-Hybrid System 3 (Clontech) as per the manufacturer’s instructions. The full-length coding sequences of *PRMT6*, *NF-YC3*, *NF-YC4*, *NF-YC9*, *NF-YB3*, *CO*, and *FT* were cloned into the *pGADT7* and *pGBKT7* vectors and subsequently introduced into the yeast strain *AH109*. Yeast cells were spotted on selective media lacking leucine (L), tryptophan (W), histidine (H), and adenine (A) for interaction detection or on drop-out media lacking L and W (as control).

### Bimolecular fluorescence complementation (BiFC) assay

The full-length coding sequences for *PRMT6*, *NF-YC3*, *NF-YC9*, and *NF-YB3* were fused with the coding sequence for an N-terminal EYFP fragment in the nEYFP-N1/pUGW0 (P_35S_/N-nEYFP) vector and/or for a C-terminal EYFP fragment in the cEYFP-N1/pUGW0 (P_35S_/N-cEYFP) vector (Nakagawa et al. 2007). Plasmid pairs were assessed by a transient expression system using *Arabidopsis* mesophyll protoplast (Yoo et al. 2007). Within 12–18 h, the EYFP fluorescence emitted from the *Arabidopsis* mesophyll protoplast was imaged with a Leica TCS SP8 laser scanning confocal microscope (Leica).

### Co-immunoprecipitation (Co-IP) assay

Co-immunoprecipitation (Co-IP) experiments were carried out as previously described with some modifications (2013). Briefly, plasmid pairs were performed by a transient expression system using *Arabidopsis* mesophyll protoplast (Yoo et al. 2007). After 12–18 h incubation, total proteins were extracted from *Arabidopsis* mesophyll protoplasts and immunoprecipitated with anti-FLAG M2 affinity gel (Sigma, Cat#: A2220), and the immunoprecipitated protein was detected by western blotting with anti-FLAG (Sigma, Cat#: A8592).

### Chromatin immunoprecipitation (ChIP) and ChIP-qPCR analysis

ChIP experiments were carried out as previously described (Gu et al. 2013). Briefly, total chromatin was extracted from 10-day-old seedlings grown under LD conditions and immunoprecipitated with anti-H3R2me2a (Abcam, Cat#: ab175007) and anti-PRMT6 (synthesized in this study). Quantitative PCR (qPCR) was conducted to measure the amounts of *FT* and the constitutively expressed *TUB8* fragments on a Roche Light Cycler 480 II System using SYBR Green PCR master mix. The ChIP-qPCR primer pairs for *FT*, were described previously (Gu et al. 2013).

### Accession numbers

Sequence data from this paper can be found in The *Arabidopsis* Information Resource (TAIR) website (http://www.arabidopsis.org/) under the following accession numbers: *PRMT6*, At3g20020; *CO*, At5g15840; *FT*, At1g65480; *NF-YC3*, At1g54830; *NF-YC4*, At5g63470; *NF-YC9*, At1g08970; *NF-YB2*, At5g47640; *NF-YB3*, At4g14540.

## Supplementary Information

Below is the link to the electronic supplementary material.

Supplementary file1 (XLSX 12 kb)

Supplementary file2 (PDF 396 kb)

## Data Availability

Not applicable.
