# Correction to: PRMT6 physically associates with nuclear factor Y to regulate photoperiodic flowering in *Arabidopsis*

**DOI:** 10.1007/s42994-021-00066-x

**Published:** 2021-12-24

**Authors:** Pingxian Zhang, Xiulan Li, Yifan Wang, Weijun Guo, Sadaruddin Chachar, Adeel Riaz, Yuke Geng, Xiaofeng Gu, Liwen Yang

**Affiliations:** 1 Biotechnology Research Institute, Chinese Academy of Agricultural Science, Beijing, 100081 China; 2 College of Life Science and Technology, Huazhong Agricultural University, Wuhan, 430070 Hubei China; 3 College of Life and Environmental Sciences, Minzu University of China, Beijing, 100081 China

## Correction to: aBIOTECH 10.1007/s42994-021-00065-y

In this article Figs. 2 and 3 were wrongly numbered; Fig. 2 should have been Fig. 3 and vice versa as shown below. Moreover, the Fig. 3 indications have been revised as shown: the sentence “Next, two transfer DNA (T-DNA) insertion single- mutant *prmt6-1* (Sail_385_A06) and *prmt6-2* (Salk_151679C) (Figs. 2A; S3a)...” has been revised as “Next, two transfer DNA (T-DNA) insertion single- mutant *prmt6-1* (Sail_385_A06) and *prmt6-2* (Salk_151679C) (Figs. 3A; S3a)...”; And the sentence “...was significantly more than that of *nf-yc3;4;9* (Fig. 2B, D)...” has been revised as “...was significantly more than that of *nf-yc3;4;9* (Fig. 3B, D)...”; And the sentence “...lower than that of the *nf-yc3;4;9* triple mutant line at ZT16 under LDs (Fig. 2E)... ” has been revised as “...lower than that of the *nf-yc3;4;9* triple mutant line at ZT16 under LDs (Fig. 3E)...”. The original article has been corrected.

**Fig. 2:**
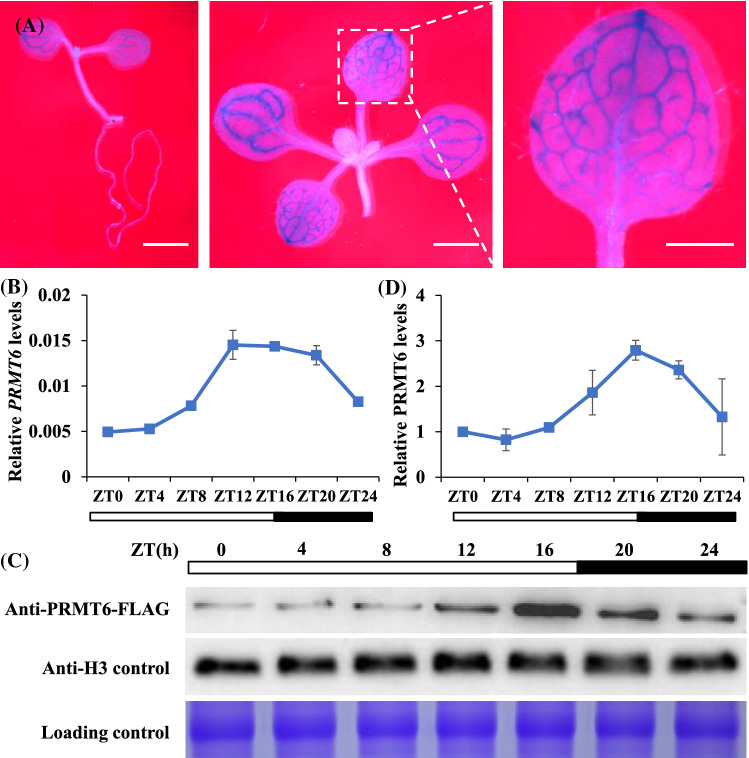
*PRMT6* diurnally expressed at dusk in the vascular bundle cells under LDs. **A** Spatial expression patterns of *PRMT6-GUS* in 5-day-old seedlings, and 10-day-old seedlings of aerial part and cotyledon. Plants were stained for 6 h. Scale bars = 1 mm. **B** The relative transcription level of *PRMT6* in 10-day-old Col seedlings under LDs. The transcription levels were normalized to *UBQ10*, and relative fold changes to Zeitgeber time 0 (ZT0) are presented. Bars indicate s.d. of triplicate measurements. White and dark bars below the -axis indicate light and dark periods, respectively. **C**, **D** The expr*x*ession levels of PRMT6-FLAG protein over a 24-h LD cycle examined by western blotting. Total proteins loaded in SDS-PAGE gels were stained with Coomassie Blue, antibody or the relative PRMT6-FLAG protein levels were normalized to H3 by the ImageJ program (**D**). The error bars indicate the s.d. measurements

**Fig. 3:**
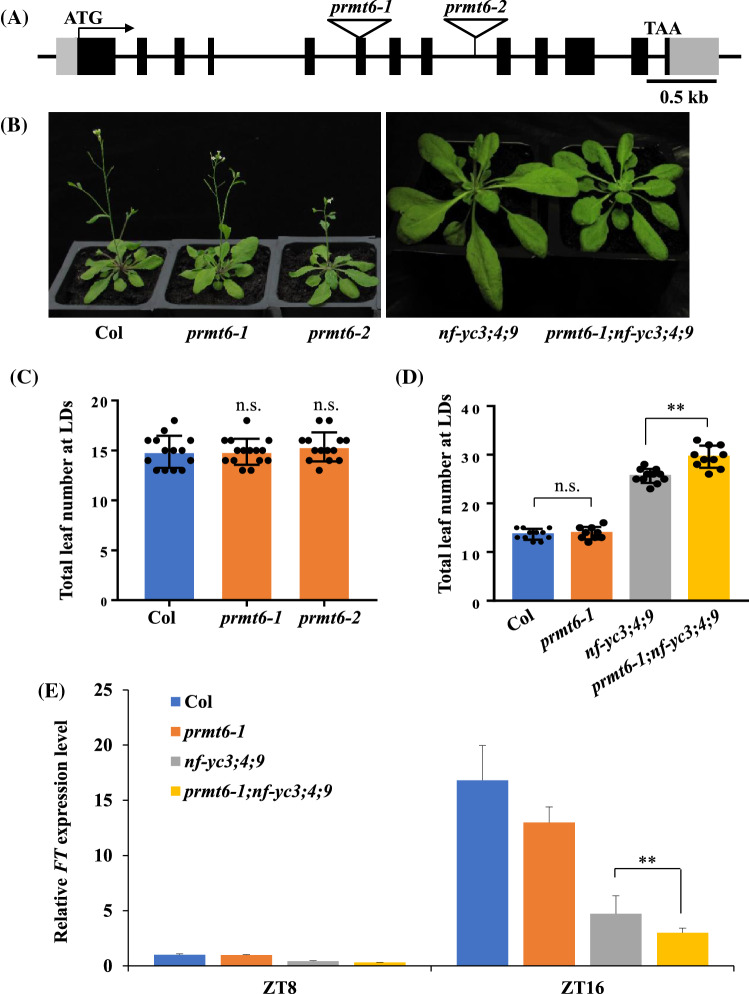
Loss of *PRMT6* function delays the floral transition of *nf-yc3;4;9* by decreasing the *FT* expression under LDs. **A** Gene structure of *PRMT6*. Exons and 5’ untranslated region (UTR) or 3’ UTR are represented by black boxes and gray boxes, and arrows indicate transcription start sites (TSS); the T-DNA insertion sites of two lines are indicated with triangles. **B** Phenotype of Col, *prmt6-1*, *prmt6-2*, *nf-yc3;4;9*, and *prmt6-1;nf-yc3;4;9* mutants grown in LDs. **C** Flowering times of the indicated genotypes grown in LDs. More than ten plants for each line were scored; bars indicated for standard deviation (s.d.); n.s. indicated non-significant difference. **D** Flowering times of the indicated lines grown in LDs. More than ten plants for each line were scored; bars indicated for s.d.; n.s. indicated non-significant difference; Double asterisks indicated statistically significant differences in the means between the indicated genotypes, as revealed by two-tailed Student’s *t* test (**p < 0.01). **E** Relative *FT* transcript levels in the seedlings of the indicated genotypes grown in LDs at ZT8 and ZT16. The transcript levels were first normalized to that of *UBQ10*. Bars indicate the s.d. of triplicate measurements

