# Supplementary material for: PRMT6 physically associates with nuclear factor Y to regulate photoperiodic flowering in Arabidopsis
Source: aBIOTECH. 2021 Dec 2;2(4):403–14. doi: 10.1007/s42994-021-00065-y (PMC9590495; doi:10.1007/s42994-021-00065-y)
Supplement: Supplementary file 2 — Supplementary file2 (PDF 396 kb) [file 42994_2021_65_MOESM2_ESM.pdf]

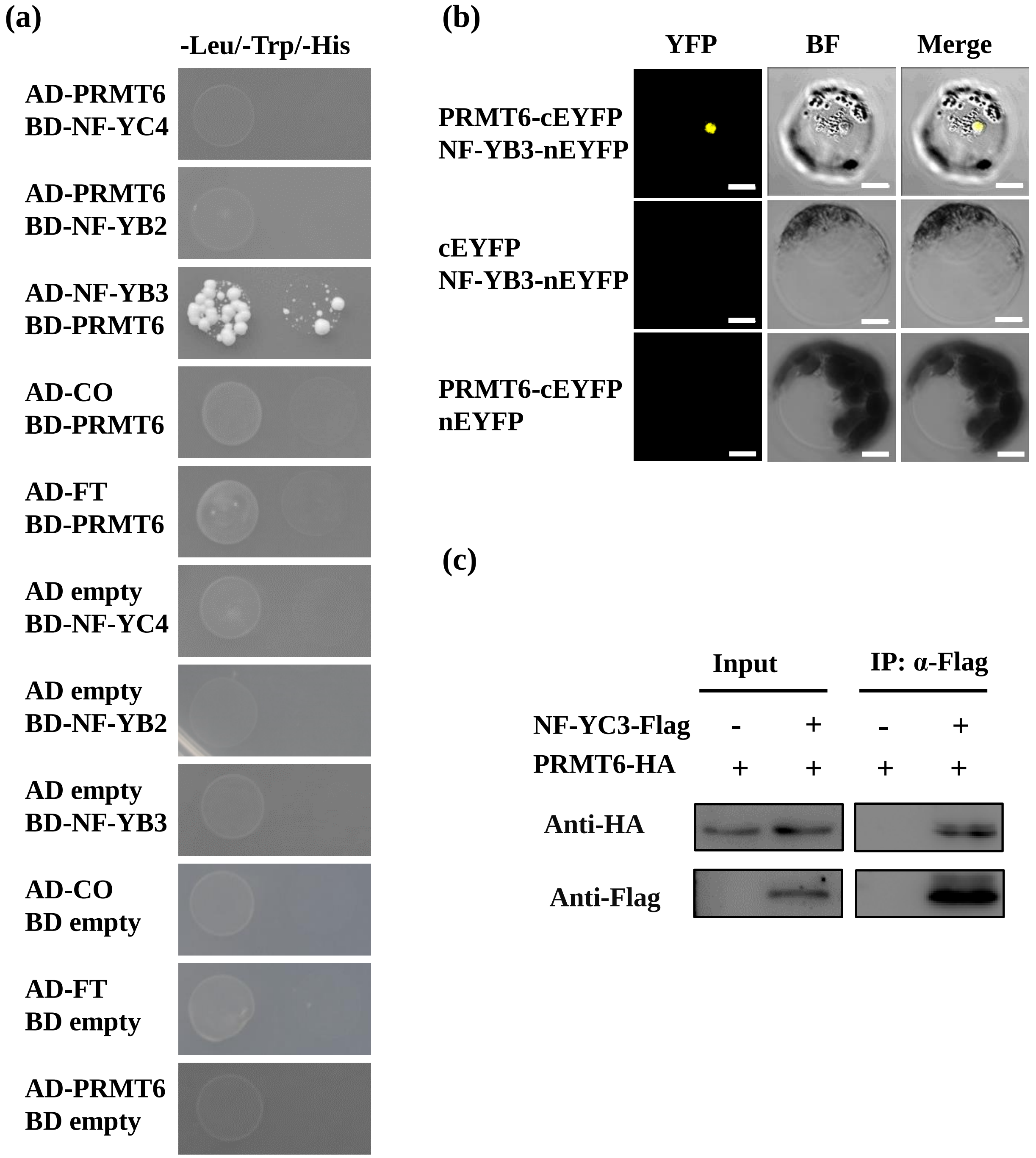


**Figure S1. Direct interaction of PRMT6 with NF-YB3 but not NF-YC4, NF-YB2, CO and FT.**

(a) Y2H assays of PRMT6 with NF-YC4, NF-YB2, NF-YB3, CO and FT. Yeast cells harboring the fusion proteins, BD and/or AD (as indicated), were grown on selective synthetic defined media lacking of Trp, Leu, and His.
(b) BiFC analysis of the interactions of PRMT6 with NF-YB3 in *Arabidopsis* protoplast. Arabidopsis protoplast were co-transformed transiently by a pair of plasmid as indicated. Yellowish-green signals indicate physical associations of paired proteins in the nuclei. Bar=10 µm.
(c) Co-IP confirmed the interaction of PRMT6 with NF-YC3.

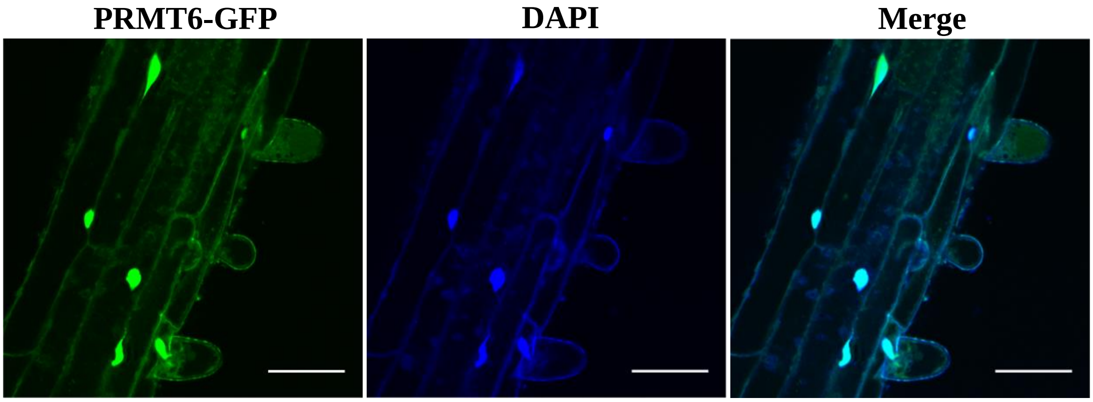


**Figure S2. Nuclear localization of the PRMT6-GFP fusion protein in roots of transgenic *Arabidopsis* seedlings.**

Scale bars = 50 μm. The blue DAPI (4',6-diamidino-2-phenylindole) staining indicates nuclei. PRMT6-GFP and DAPI fluorescence were imaged using a laser scanning confocal microscope.

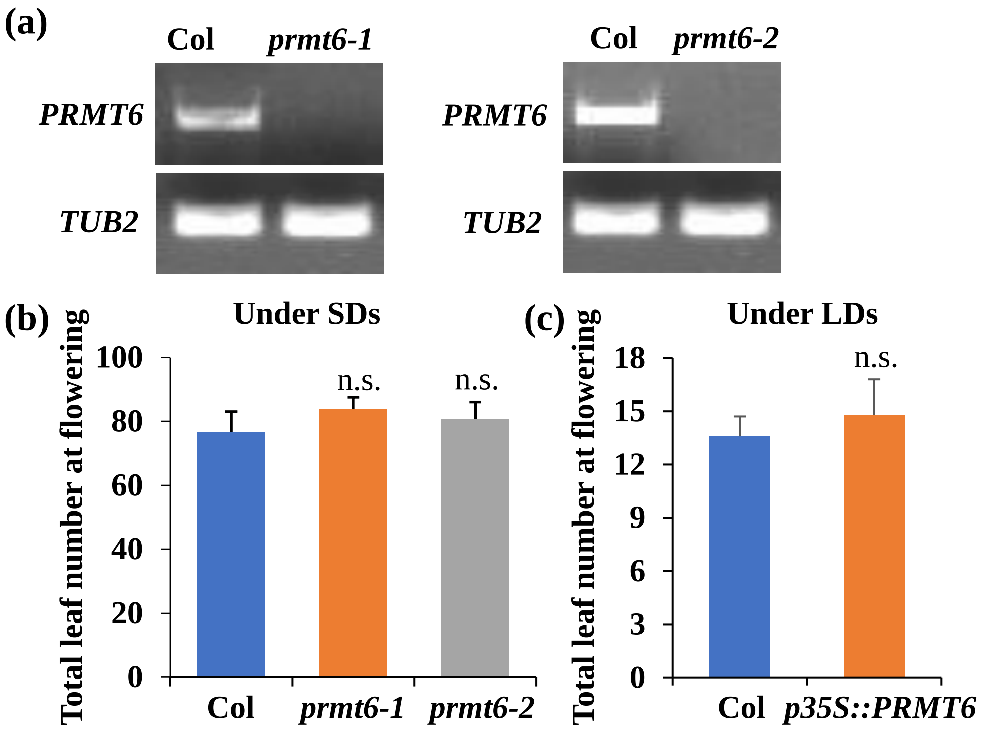


**Figure S3. The identification and flowering time of *prmt6* mutants.**

(a) RT-PCR analysis of full length *AtPRMT6* in Col and mutant lines. *TUBULIN 2* was used as a control.
(b) Flowering time of *prmt6* mutants grown under SDs. More than 10 plants for each line were scored; bars indicated for standard deviation (s.d.)
(c) Flowering time of *PRMT6* overexpression lines grown under LDs.

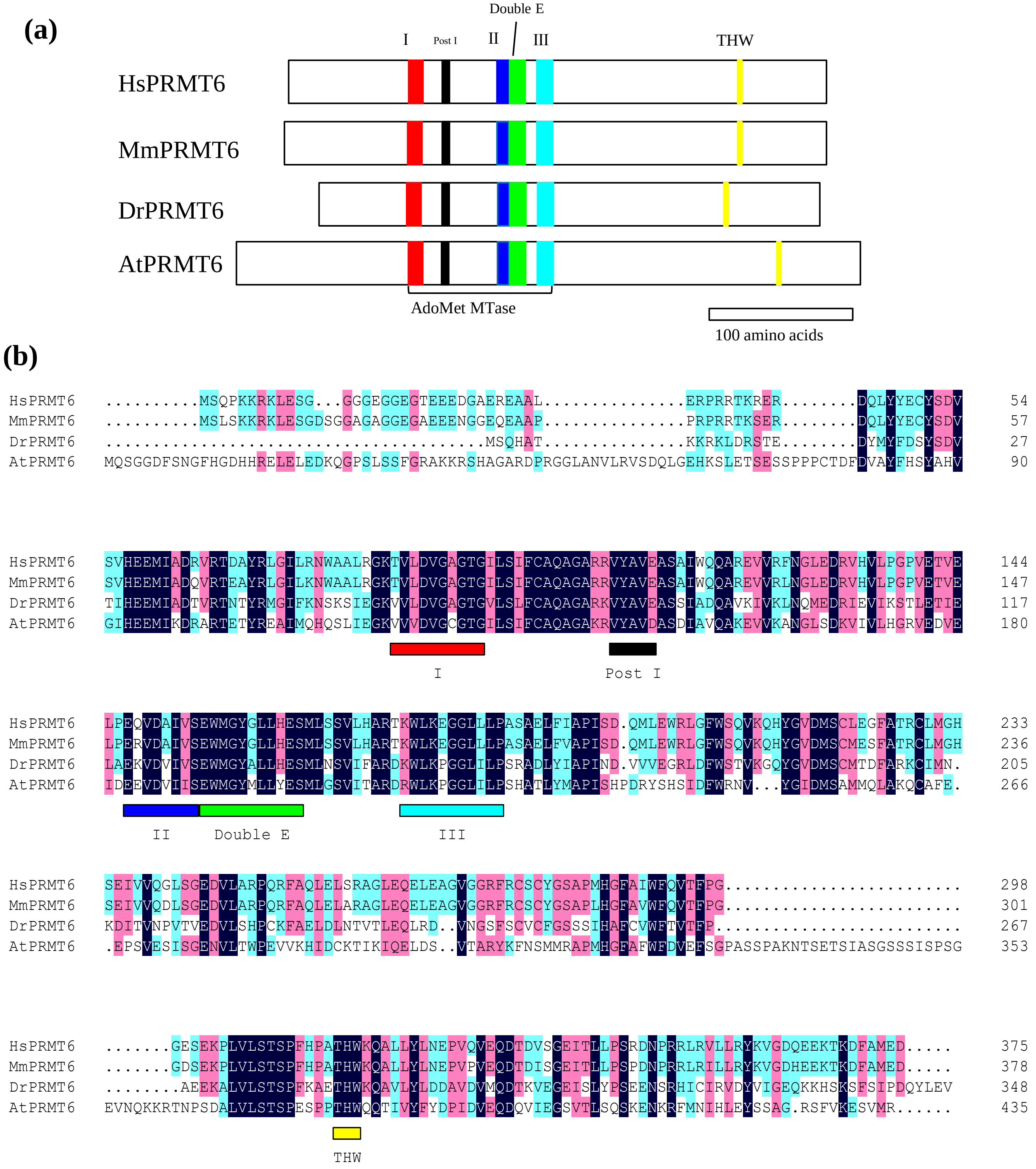


**Figure S4. Conserved functional domains of *Arabidopsis* (AtPRMT6), human (HsPRMT6), zebrafish (DrPRMT6) and mouse (MmPRMT6).**

(a) Domain structures of PRMT6 proteins. Motifs I, post-I, II, double-E loop, III and THW loop were indicated in different color. The AdoMet MTase domain was indicated at bottom.

(b) Amino acid sequence alignment of PRMT6 proteins from *Arabidopsis*, rice, human, zebrafish and mouse. The conserved methyltransferase motifs I, post-I, II, double-E loop, III and THW loop were indicated with different color lines. Sequence were obtained from UniProt (www.uniprot.org/).

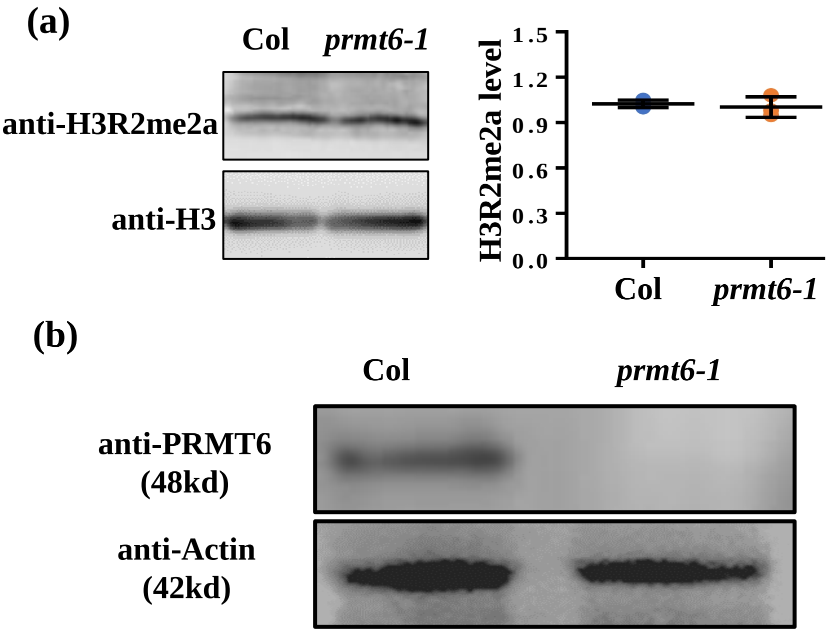


**Figure S5. Western blot analysis the expression of PRMT6 and H3R2me2a in Col and *prmt6-1*.**

(a) The identification of PRMT6 antibody. (b) the H3R2me2a level in Col and *prmt6-1* seedlings. Total proteins extracted from 10-d old seedlings of the indicated genotypes. PRMT6 antibody was constructed from Beijing Enris Biotechnology Co. Actin antibody was used as a control. For H3R2me2a methylation level, band intensities of triplicate technical repeats were quantified by the ImageJ program. The error bars are the s.d. from three replicates.

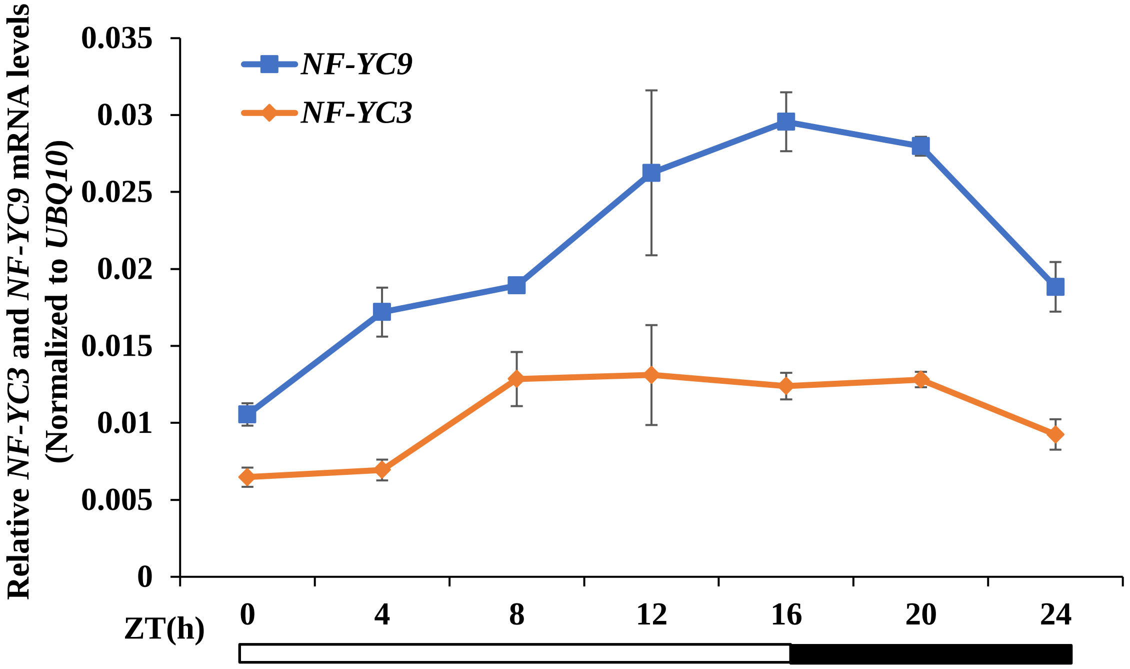


**Figure S6. *NF-YC3* and *NF-YC9* exhibit a diurnal patterns under LDs.**

The relative transcription levels of *NF-YC3* and *NF-YC9* in 10-d-old Col seedlings under LDs. The transcription levels were normalized to *UBQ10*. Bars indicate s.d. from triplicate measurements. White and dark bars below the x-axis indicate light and dark periods respectively.
